# The structure-function relationship measured with optical coherence tomography and a microperimeter with auto-tracking: the MP-3, in patients with retinitis pigmentosa

**DOI:** 10.1038/s41598-017-16143-5

**Published:** 2017-11-17

**Authors:** Yuichi Asahina, Marie Kitano, Yohei Hashimoto, Mieko Yanagisawa, Hiroshi Murata, Tatsuya Inoue, Ryo Obata, Ryo Asaoka

**Affiliations:** 1 Department of Ophthalmology, The University of Tokyo, Tokyo, Japan; 2 Department of Ophthalmology, the University of Tokyo, Graduate School of Medicine, 7-3-1 Hongo, Bunkyo-ku, Tokyo, Japan

## Abstract

The purpose of the current study was to investigate the structure-function relationship in patients with retinitis pigmentosa (RP) using optical coherence tomography and the MP-3 microperimeter. Visual field (VF) measurements were carried out using MP-3 microperimetry and the Humphrey Field Analyzer (HFA, Carl-Zeiss, CA), 22 eyes of 11 patients with a clinical diagnosis of RP, both with the 10-2 test grid pattern. Optical coherence tomography (OCT, Spectralis, Heidelberg, Germany) was also performed and the ellipsoid zone (EZ) was identified in the OCT image. The mean (±SD) number of test points located within the EZ edge was 11.6 (±5.9). There was a significant relationship between mean retinal sensitivity measured with MP-3 and the area surrounded by the EZ circular line: AEZ (p < 0.05), but this was not the case with HFA (p > 0.05). The difference between retinal sensitivity inside and outside the EZ edge was significantly larger with MP-3 than with HFA (p < 0.001). Our findings suggest that retinal sensitivity measured with MP-3 better reflects the magnitude of structural damage observed with OCT, compared with HFA. Further, the difference in retinal sensitivity between the inside and outside EZ edge is significantly larger for the MP-3 test, compared with the HFA.

## Introduction

Retinitis pigmentosa (RP) is a progressive retinal disease characterized by a loss of photoreceptors^1,2^. Damage to the rod photoreceptors is associated with nyctalopia. The cone photoreceptors are also involved, usually at a later stage of the disease, causing loss of visual function, such as constriction of the visual field (VF). VF damage usually begins in the peripheral area, but spreads toward the central area as the disease progresses, which can result in deterioration of visual acuity (VA).

In RP patients, the assessment of VF damage is usually performed using a static automated perimeter (SAP), such as the Humphrey Field Analyzer (HFA, Carl Zeiss Meditec AG, Dublin, CA, USA). Measurement noise, however, is hugely problematic with VF testing, hampering the diagnosis of RP and then the detection of its progression^3,4^. There are many different causes for the variability associated with VF measurements, but eye movements during the test are closely related to unreliable results and under-estimation of VF sensitivity^5,6^. To help overcome this issue, the new MP-3 microperimeter (Nidek co.ltd, Aichi, Japan) is equipped with an auto-tracking function; the position of the retina is accurately followed throughout the VF test so that the target stimulus is projected onto a precise location on the retina. The MP-3 also has a much wider dynamic range (between 0 and 34 dB) than its predecessor, the MP-1. The background luminance in the MP-3 is 31.4 asb, which is identical to that in the HFA. We recently reported that VF sensitivity measured with the MP-3 is associated with significantly better test-retest reproducibility compared to the HFA, in patients with RP^7^.

In this study, we assessed structural damage in RP patients, using optical coherence tomography, by measuring the area of the remaining ellipsoid zone (EZ), and explored the structure-function relationship between MP-3 and HFA VF measurements.

## Method

This study was approved by the Research Ethics Committee of the Graduate School of Medicine and Faculty of Medicine at The University of Tokyo. Written informed consent was given by patients for their information to be stored in the hospital database and used for research. This study was performed according to the tenets of the Declaration of Helsinki.

### Subjects

This study included 22 eyes (11 right and 11 left eyes) of 11 patients with RP (4 males and 7 females), diagnosed based on clinical examination, VF measurements, and electroretinography. All patients were prospectively recruited at the retina clinic in The University of Tokyo Hospital. All patients fulfilled the following inclusion criteria: (1) patients had a typical phenotype as RP, such as progressive concentric VF constriction, night blindness, and mid-peripheral intraretinal perivascular ‘bone-spicule’ pigmentary changes and RPE atrophy associated with arteriolar narrowing on color fundus imaging; (2) RP was the only disease causing VF damage; (3) patients were followed for at least 6 months at The University of Tokyo Hospital and underwent at least two VF measurements prior to this study; (4) measured best- corrected visual acuity was above 20/40; (5) pupil size was larger than 4 mm in diameter, which is required for the MP-3 measurement; (6) a ring shaped abnormal hyperfluorescent perifoveal ring was observed within the central 10 degrees on fundus autofluorescence imaging.

### 10-2 VF measurements

Each patient underwent VF testing twice with the HFA (10-2 Swedish Interactive Threshold Algorithm, SITA, standard program) and the MP-3 microperimeter, within a three month period, in different days. The order of the HFA or MP-3 measurement was decided in a random manner. A white-on-white HFA 10-2 measurement was carried out with the Swedish Interactive Threshold Algorithm Standard (SITA) test and the standard Goldmann III stimulus size. Only reliable VFs were used in the analyses, defined as a fixation loss (FL) rate <20% and a false-positive (FP) rate <15%, but a false negative (FN) rate was not used as an exclusion criterion, following the criteria used by the manufacturer.

**Figure 1:**
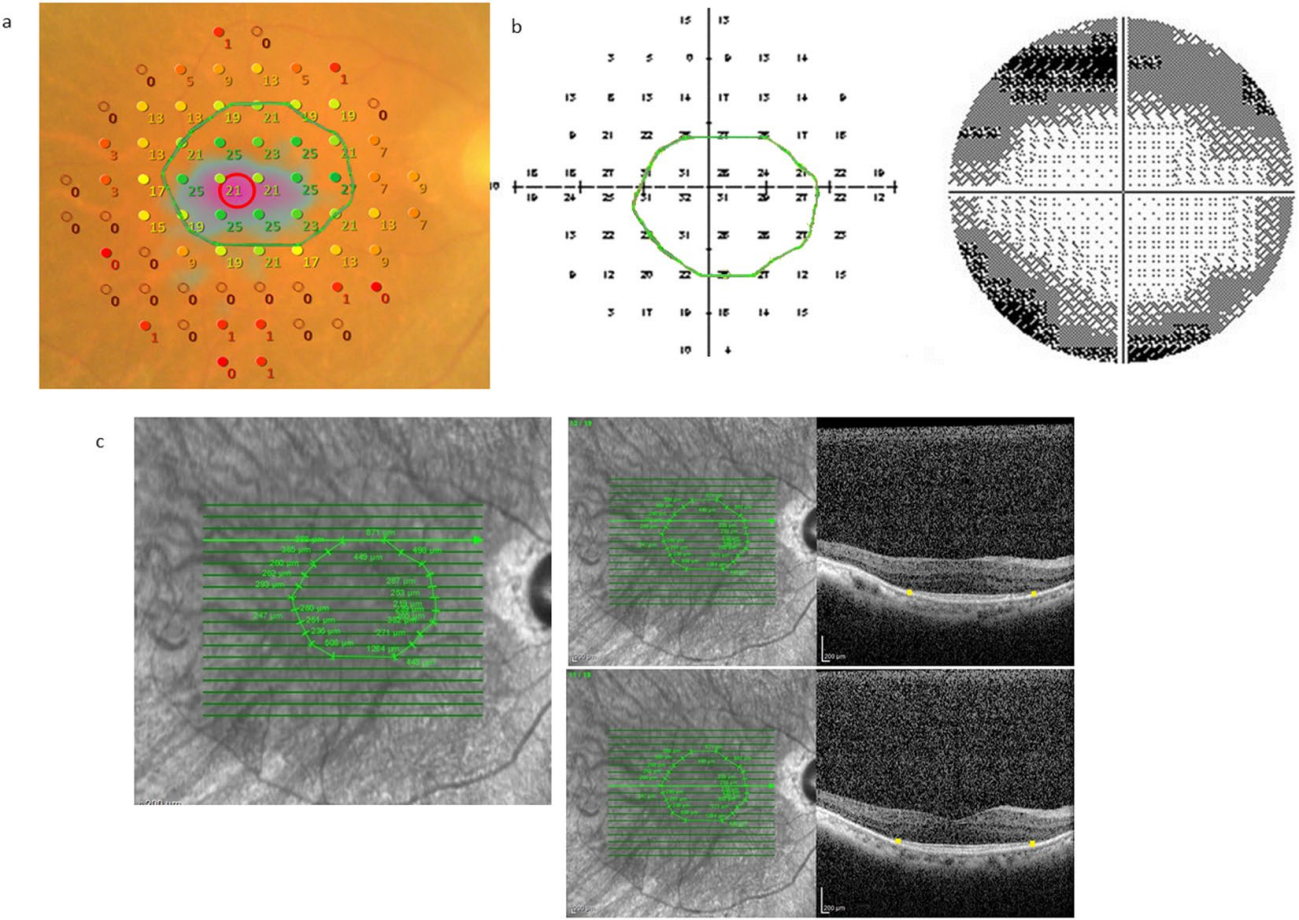
Example VF measurement with the MP-3 and the HFA, and identified EZ circular line. Perimetry results of (**a**) 69 year old female with RP, for the differential light thresholds with (**a**) MP-3 (MP-3 results were flipped vertically) and (**b**) HFA. (**c**) EZ ellipsoid zone identification was carried out using each slice of the cross-sectional OCT image. EZ: ellipsoid zone, VF: visual field, HFA: Humphrey Field Analyzer, RP: retinitis pigmentosa, OCT: optical coherence tomography.

The details of the MP-3 measurement are detailed elsewhere^7^. In short, the MP-3 measurement is based on a 4-2 full threshold staircase strategy, similarly to the HFA. The stimulus dynamic range is between 0 and 34 dB which is narrower than HFA, however the sensitivity within this dynamic range can be directly compared to that with HFA, because the background luminance is 31.4 asb and the maximum brightness of the stimulus of 10,000 asb, which are identical to those in HFA. The MP-3 measurement was carried out using the standard Goldmann III stimulus size and a measured test grid identical to the HFA 10-2 test pattern (Fig. 1). Once again, only reliable VFs were used in analyses: a fixation loss (FL) rate <20% and a false-positive (FP) rate <15%. After completion of the sensitivity testing, a color fundus image with the superimposed sensitivity values was exported from the device.

### Optical coherence tomography measurement

Spectral domain (SD) OCT data were obtained using the Spectralis OCT (Heidelberg Engineering, Heidelberg, Germany). All OCT images consisted of line scans (horizontal and vertical B-scans), raster scans, and topographic mapping. Line scans were created by averaging up to 100 B-scans (768 A-scans per B-scan) within 30°. The raster scan was performed using 25 B-scans (768 A-scans per B-scan) of a 30°x20° area. The line scans, raster scans, and topographic mapping were performed on each eye. Each measurement was segmented into 19 images. With these 19 images, the remaining EZ was identified and recorded in each patient and the boundary was defined as the ‘EZ edge’ (Fig. 1). VF test points with MP-3 and also HFA 10-2 were classified into two groups; inside the outer EZ edge, and outside the EZ edge. If the outer EZ edge completely matches a test point, the test point was classified as outside the EZ. Then, the en-face OCT images, with the defined EZ edge, were superimposed onto the MP-3 color fundus images. In Fig. 1, these OCT images were also superimposed onto HFA 10-2 test results, but in an upside-down manner, because the superior area on the fundus is relevant to the inferior hemifield in the visual field. Initial identification of the EZ line was performed by an examiner (YA) followed by verification by an independent examiner (MK). If the second estimator did not agree with the first examiner, a panel discussion was held and the final EZ line was decided.

The area surrounded by the EZ circular line (AEZ) was automatically calculated by tracing the EZ circular line in the ImageJ software (version 1.48, http://imagej.nih.gov/ij/; provided in the public domain by the National Institutes of Health, Bethesda, MD, USA).

### Statistical analysis

The relationship between AEZ and the mean sensitivity measured with HFA and MP-3 was calculated using a linear mixed model, whereby each eye is nested within each patient. The linear mixed model is equivalent to ordinary linear regression in that the model describes the relationship between the predictor variables and a single outcome variable. However, standard linear regression analysis is based on the assumption that all observations are independent of each other. In the current study, measurements are dependent on each other, because they are nested within subjects. Ignoring this structure of the dataset results in the underestimation of standard errors of regression coefficients. In contrast, the linear mixed model adjusts for the hierarchical structure of the data, whereby measurements are grouped within subjects. Mean sensitivity was calculated using the linear sensitivity at each test point for both the HFA and the MP-3. In this calculation, retinal sensitivity at each test point was first converted from (dB) to (1/Lambert), and the average value was calculated. Then the average linear retinal sensitivity was converted back to the (dB) scale.

The mean retinal sensitivities inside and outside the EZ edge were calculated and the difference between the mean of retinal sensitivities inside the outer EZ edge, and outside the EZ edge was evaluated (there was no test points located exactly on the outer EZ edge). Then the magnitude of the difference was compared between MP-3 and HFA perimeters, using the linear mixed model, whereby each eye was nested within each patient. Marginal R-squared (mR^2^) value was calculated following a method proposed by Nakagawa and Holger^8^. In the linear mixed model, both fixed and random effects are analyzed. The mR^2^ is calculated based on only fixed effect.

All analyses were performed using the statistical programming language ‘R’ (R version 3.1.3; The Foundation for Statistical Computing, Vienna, Austria).

## Result

Subject demographics are shown in Table 1. With the first VF, the duration of the MP-3 measurement was 15 minutes and 49 seconds ± 3 minutes and 19 seconds [10 minutes and 51 seconds to 20 minutes and 44 seconds] (mean ± standard deviation: SD [range]), which was significantly (p < 0.001, linear mixed model) longer than the HFA measurement (7 minutes and 14 seconds ± 12 minutes and 6 seconds [5 minutes and 36 seconds to 10 minutes and 13 seconds]). With the second VF, the duration of the MP-3 measurement was 16 minutes and 28 seconds ± 3 minutes and 32 seconds [11 minutes and 8 seconds to 21 minutes and 22 seconds] (mean ± standard deviation: SD [range]), which was significantly (p < 0.001, linear mixed model) longer than the HFA test (7 minutes and 14 seconds ± 12 minutes and 6 seconds [5 minutes and 32 seconds to 9 minutes and 15 seconds]).

**Table 1:** Subject demographics.

variables	value
age, years old, mean ± sd [range]	44.8 ± 15.4 [21 to 69]
gender, male:female	4:7
mean retinal sensitivity with 1st HFA, dB, mean ± sd [range]	21.0 ± 3.7 [13.7 to 28.1]
mean retinal sensitivity with 1st MP-3, dB, mean ± sd [range]	11.0 ± 3.5 [4.8 to 17.6]
mean lienar retinal sensitivity with 1st HFA, dB, mean ± sd [range]	25.7 ± 2.6 [20.1 to 30.6]
mean linear retinal sensitivity with 1st MP-3, dB, mean ± sd [range]	18.0 ± 3.4 [10.2 to 23.8]
mean retinal sensitivity with 2nd HFA, dB, mean ± sd [range]	20.7 ± 3.5 [12.8 to 27.3]
mean retinal sensitivity with 2nd MP-3, dB, mean ± sd [range]	10.6 ± 2.2 [6.2 to 17.3]
mean lienar retinal sensitivity with 2nd HFA, dB, mean ± sd [range]	25.6 ± 2.5 [20.1 to 29.8]
mean linear retinal sensitivity with 2nd MP-3, dB, mean ± sd [range]	17.7 ± 3.7 [10.8 to 24.0]
S_EZ_, mm^2^, mean ± sd [range]	4.3 ± 2.4 [0.17 to 8.0]

EZ: ellipsoid zone, HFA: Humphrey afield Analyzer, SD: standard deviation, AEZ: area surrounded by the EZ circular line.

**Figure 2:**
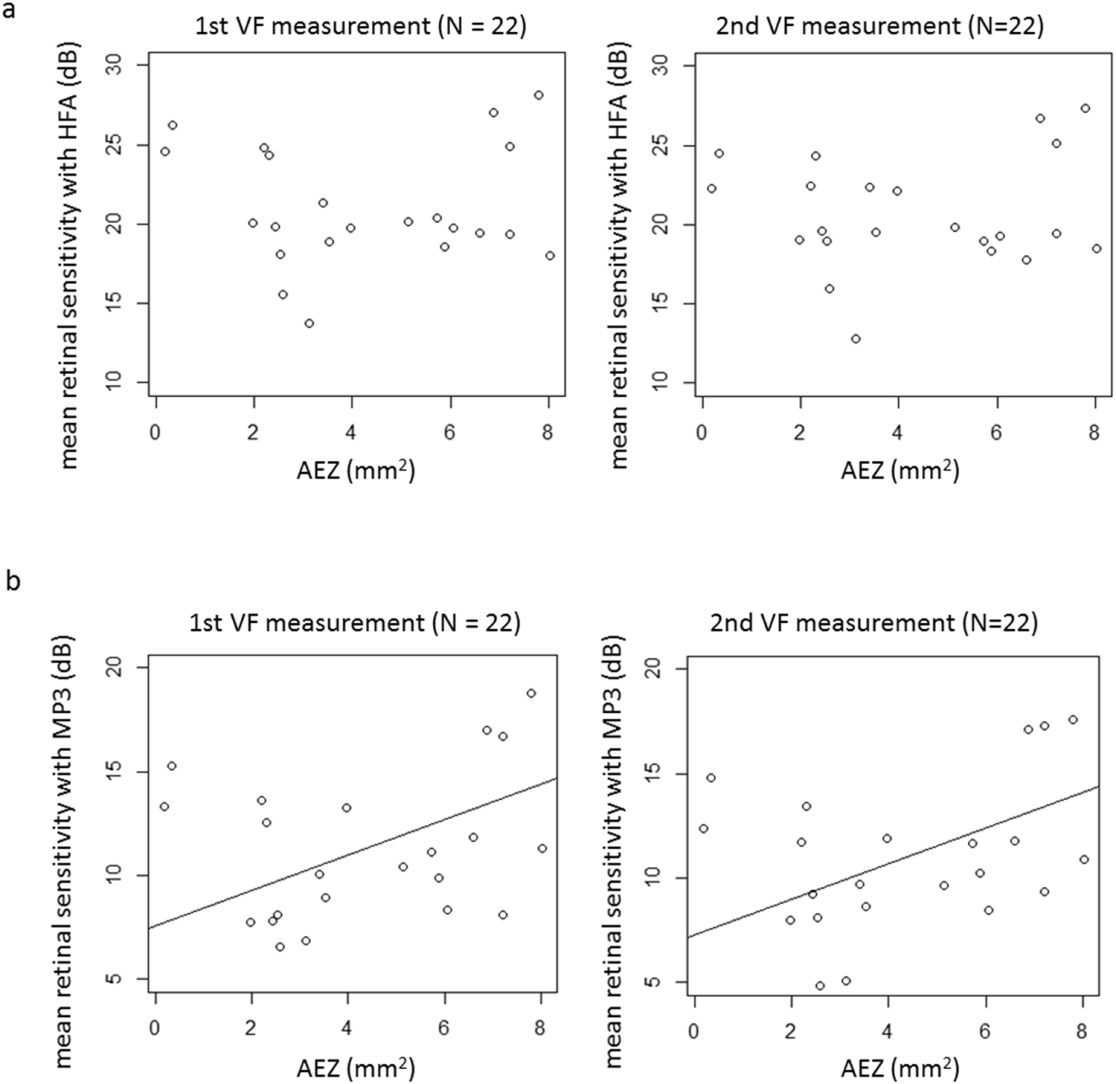
The relationship between mean retinal sensitivity with the HFA and the MP-3, and AEZ. Mean retinal sensitivity was calculated by averaging retinal sensitivity on the dB scale. (**a**) HFA. There was no significant relationship between mean retinal sensitivity with the first and second HFA tests and AEZ (p = 0.30 and 0.29, respectively, linear mixed model), (**b**) MP-3. With the first MP-3, mean retinal sensitivity was significantly related to AEZ: MP-3 retinal sensitivity = 7.6 + 0.85 × AEZ (p = 0.041, linear mixed model); with the second MP-3, mean retinal sensitivity was also significantly related to AEZ: 7.3 + 0.85 x AEZ (p = 0.042, linear mixed model). EZ: ellipsoid zone, VF: visual field, HFA: Humphrey Field Analyzer, AEZ: area surrounded by the EZ circular line.

Figure 2 shows the relationship between AEZ and mean sensitivity (dB) measured with HFA and MP-3. With both the first and second HFA test results, mean retinal sensitivities (dB) were not significantly related to the AEZ (p = 0.30, mR^2^ = 0.053: 1st HFA and p = 0.29: 2nd HFA, mR^2^ = 0.070, linear mixed model). With the first MP-3 test, mean retinal sensitivity (dB) was significantly related to AEZ: MP-3 retinal sensitivity (dB) = 7.6 + 0.85 x AEZ (p = 0.041, mR^2^ = 0.23, linear mixed model). For the second MP-3 test, mean retinal sensitivity (dB) was again significantly related to AEZ: MP-3 retinal sensitivity (dB) = 7.3 + 0.85 x S_EZ_ (p = 0.042, mR^2^ = 0.24, linear mixed model).

**Figure 3:**
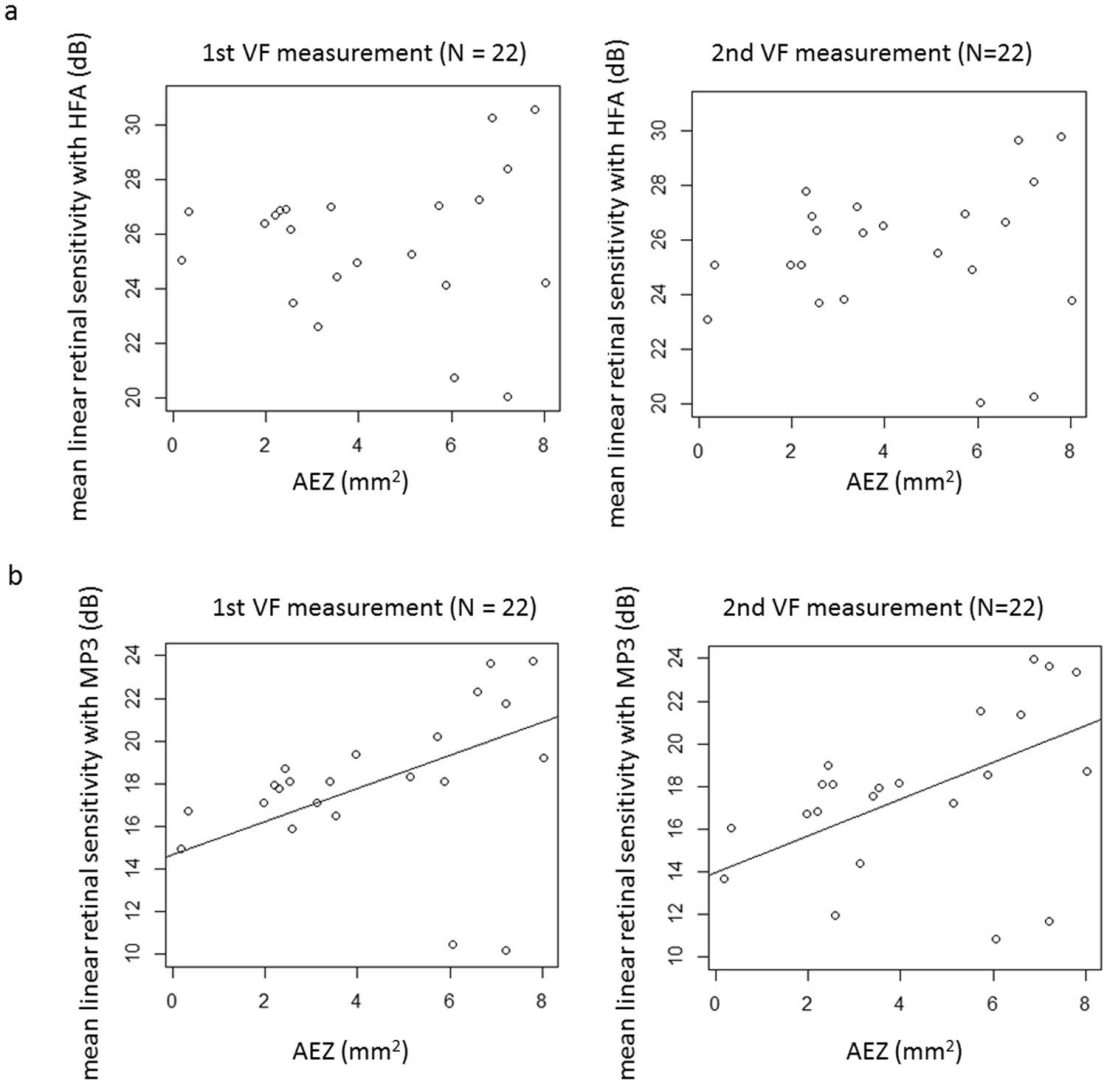
The relationship between mean linear retinal sensitivity with the HFA and the MP-3, and AEZ. (**a**) HFA. With both the first and second VFs, mean retinal sensitivity was not significantly related to AEZ (p = 0.17 and 0.66, respectively, linear mixed model). (**b**) MP-3. With the first VF, mean retinal linear sensitivity (dB) was significantly related to AEZ: MP-3 retinal sensitivity = 14.6 + 0.78 x AEZ (p = 0.022, linear mixed model); with the second VF, mean retinal linear sensitivity (dB) was significantly related to AEZ: MP-3 retinal sensitivity (dB) = 14.0 + 0.86 x area surrounded by EZ circular line (p = 0.032, linear mixed model). EZ: ellipsoid zone, VF: visual field, HFA: Humphrey Field Analyzer, AEZ: area surrounded by the EZ circular line.

Figure 3 shows the relationship between AEZ and mean linear retinal sensitivity (dB) measured with HFA and MP-3. With the first and second VFs, mean retinal sensitivity (dB) measured with the HFA was not significantly related to AEZ (p = 0.17, mR^2^ = 0.078 and p = 0.40, mR^2^ = 0.22, respectively, linear mixed model). With the first and second VFs, mean retinal sensitivity (dB) measured with the MP-3 was significantly related to AEZ: MP-3 retinal sensitivity (dB) = 14.6 + 0.78 x AEZ (p = 0.022, mR^2^ = 0.070, linear mixed model): first VF, MP-3 retinal sensitivity (dB) = 14.0 + 0.86 x AEZ (p = 0.032, mR^2^ = 0.24, linear mixed model): first VF.

**Figure 4:**
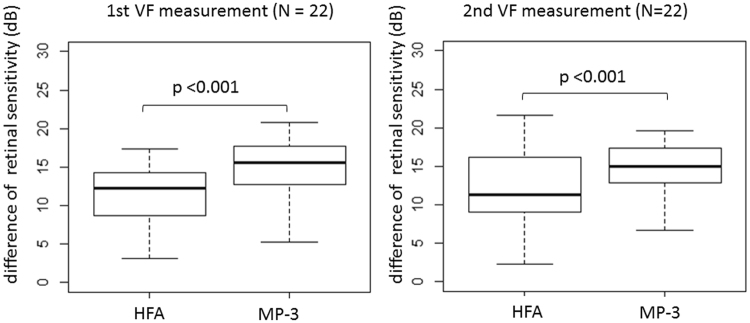
Boxplot comparing mean retinal sensitivity outside and inside the EZ circular line. The difference between mean retinal sensitivity outside and inside EZ circular line was significantly larger with the MP-3 than with the HFA (p < 0.001, linear mixed model). EZ: ellipsoid zone, VF: visual field, HFA: Humphrey afield Analyzer.

There were 11.6 ± 5.9 (mean ± SD) test points located within the EZ edge. As shown in Fig. 4, with the first VFs, the mean retinal sensitivity inside the EZ edge was 23.4 ± 4.0 and 30.4 ± 3.5 dB, with the MP-3 and HFA, respectively. Mean retinal sensitivity outside the EZ edge was 8.7 ± 3.7 and 23.4 ± 4.0 dB with the MP-3 and HFA, respectively. The difference between retinal sensitivity inside and outside the EZ edge was significantly larger with the MP-3 than with the HFA (p < 0.001, linear mixed model). With the second VFs, mean retinal sensitivity inside the EZ edge was 22.6 ± 4.1 and 30.5 ± 3.5 dB, with the MP-3 and HFA, respectively. Mean retinal sensitivity outside the EZ edge was 8.4 ± 3.6 and 18.6 ± 4.1 dB with the MP-3 and HFA, respectively. The difference between retinal sensitivity inside and outside the EZ edge was significantly larger with the MP-3 than with the HFA (p < 0.001, linear mixed model).

## Discussion

In the current study, VF measurements were carried out using the HFA and MP-3 perimeters in patients with RP. Macular OCT was also carried out and the EZ circular line was identified. Mean retinal sensitivity measured with HFA was not significantly related to AEZ, however, mean retinal sensitivity measured with the MP-3 was significantly related to AEZ. Further, AEZ was more closely related to mean VF sensitivity from the MP-3 than from the HFA. In addition, the difference between mean retinal sensitivities inside and outside the EZ edge was significantly larger with the MP-3 compared to the HFA.

In our recent study, repeat HFA and MP-3 measured sensitivity measurements were compared in patients with RP^7^. As a result, it was suggested that test-retest reproducibility was similar with both VFs, although the intraclass correlation coefficient was significantly better with the MP-3 instrument. Furthermore, retinal sensitivity measured with the MP-3 was significantly lower than that with the HFA. Similarly, in the current study, retinal sensitivity was much lower with the MP-3 (mean retinal sensitivity was 11.3 or 10.6 dB) than with the HFA (mean retinal sensitivity was 21.0 or 20.7 dB). As suggested in our previous report, reduced sensitivity in the MP-3 may be due to its prolonged measurement duration, since longer VF testing is associated with lower measured VF sensitivity^9,10^. It should be noted that different measurement strategies were used for the HFA (SITA standard) and the MP-3 (full threshold) test. However, it is unlikely that this can explain all of the observed reduction in measured sensitivity, which was approximately 10 dB in the current study. In a previous study, the sensitivity difference between the full threshold and SITA strategies was merely 3 dB^11^. In perimeters without auto-tracking, such as the HFA, small eye movements (less than 3°) during the VF measurement cannot be avoided, even in well-trained healthy subjects^12,13^. In the MP-3 test, however, the stimulus is projected onto a particular position of the retina, after adjustment for eye movements according to the perimeter’s auto-tracking function, also as discussed in our previous paper^7^. Hence retinal sensitivity is accurately measured at an exact point. This may lead to light adaptation at the test point causing retinal sensitivity to become lower at that point due to repeated target presentations. This phenomenon may be exaggerated in RP patients, because the photoreceptor cell itself is the site of disease damage in RP. In HFA, this light adaptation may not occur, because of eye movement. However, at the same time, the light target is perceived at different locations in HFA, due to the eye movement. In contrast, with MP-3, light adaptation may occur, however the light target is projected on the exact same location on retina. Thus both have potential advantage and disadvantage. In our previous paper, we reported test-retest reproducibility was better with MP-3 in patients with RP^7^. In the current study, structure-function relationship is better with MP-3 in RP. These findings would suggest the visual sensitivity with MP-3 is better describing visual function in RP. Also, the background brightness and maximum brightness of the target are identical in HFA and MP-3. However, in HFA, the magnitude of the light reaches retina is influenced by pupil size, whereas this is not the case with MP-3, because the light is narrower than pupil size. Thus, more accurate assessment of visual sensitivity may be achieved with MP-3 than with HFA, which may be another reason the visual function in RP was better assessed with MP-3. The narrower stimulus dynamic range (0 to 34 dB) of MP-3 can potentially have an effect on the results, however the maximum sensitivity with MP-3 in the current study was much less than 34 dB (31 dB), and hence it is not relevant to the current results.

Despite the narrower range of measured retinal sensitivity in the MP-3, the structure-function relationship between AEZ and mean retinal sensitivity was stronger for MP-3 than with the HFA. Rangaswamy *et al*. have investigated the relationship between the thickness of the photoreceptor outer segment and VF sensitivity, and suggested the linear model fits better when retinal sensitivity is converted to the linear scale from the dB scale^14^. In the current study, mean retinal sensitivities were calculated directly using the dB unit and via the 1/Lambert unit. As a result, HFA mean retinal sensitivities were not significantly correlated to AEZ. On the other hand, for MP-3, both mean retinal sensitivities and mean linear retinal sensitivities were significantly correlated to AEZ with both the first and second VF tests (Figs 2b and 3b).

Disappearance of the EZ is a clinical marker in the pathology of RP. The EZ disappears early on in the disease process^15–17^, and it has been reported that the EZ edge corresponds to the edge of the VF^15^. In addition, Birch *et al*. have reported that it is useful to investigate VF sensitivities inside and outside the EZ edge to detect the progression of RP^18^. However, it has also been reported that a considerable magnitude of retinal sensitivity loss (approximately 8 dB) is observed when the disappearance of EZ is detected^14^. One of the possible explanations for this gap between structure and function is the inaccuracy of VF measurements. The HFA is not equipped with auto-tracking, and, as described above, this type of VF measurement cannot avoid small, such as less than 3°, eye movements during the measurement, even in well-trained healthy subjects^12,13^, which is not negligible considering the large Goldmann size III stimulus: a diameter of 0.43 degrees. Again, despite the much narrower range of retinal sensitivity with the MP-3 compared to the HFA, the difference of retinal sensitivity between the inside and outside EZ edge was significantly larger with the MP-3 than with the HFA. This result strongly suggests that the MP-3 VF test is more sensitive to detect structural changes captured with OCT. Thus the stronger structure-function relationship was observed with MP-3 than with HFA, which would be attributed to the difference of the measurement mechanisms; MP-3 is equipped with auto-tracking which ensures to stimulate exact same location in the VF measurement.

Numerous studies have used multifocal electroretinography (mfERG) to evaluate function in RP patients^14,19–24^. Wen *et al*. reported that mfERG amplitude correlates with the remaining thickness of the photoreceptor layer, similar to visual field sensitivity^25^. A limitation of the current study is that the mfERG measurement was not carried out. A future study is needed to explore the relationship between mfERG amplitude and retinal sensitivity measured with the HFA and the MP-3. As discussed in our previous report^7^, a limitation of the current MP-3 measurement is that only a full threshold strategy is available, and, as a result, a significantly longer test duration is needed, compared to the HFA SITA standard test. Efforts should be made to enable shorter MP-3 test as this may be advantageous to improve its accurate estimation of retinal sensitivity^11^. Furthermore, considerable time is currently required for the MP-3’s auto-tracking system to align the eye’s fixation position; this will result in patient fatigue. It is important to determine how any change in this alignment affects test accuracy, striking the right balance between reproducibility of the stimulus position on the retina and shortening test duration.

In conclusion, retinal sensitivity measured with the MP-3 was significantly related to the area surrounded by the EZ circular line. This was not the case for HFA retinal sensitivity measurements. In addition, the difference between retinal sensitivities inside and outside the EZ circular line were more pronounced with the MP-3 than with the HFA.
